# Correction: DiffBP: generative diffusion of 3D molecules for target protein binding

**DOI:** 10.1039/d5sc90047f

**Published:** 2025-03-25

**Authors:** Haitao Lin, Yufei Huang, Odin Zhang, Siqi Ma, Meng Liu, Xuanjing Li, Lirong Wu, Shuiwang Ji, Tingjun Hou, Stan Z. Li

**Affiliations:** a Zhejiang University Hangzhou 310058 Zhejiang China tingjunhou@zju.edu.cn; b AI Lab, School of Engineering, Westlake University Hangzhou 310024 Zhejiang China Stan.ZQ.Li@westlake.edu.cn; c Texas A&M University Texas TX 77843 USA; d Jingdong Beijing 101111 China

## Abstract

Correction for ‘DiffBP: generative diffusion of 3D molecules for target protein binding’ by Haitao Lin *et al.*, *Chem. Sci.*, 2025, **16**, 1417–1431, https://doi.org/10.1039/D4SC05894A.

The authors regret that in the original publication the name of one of the authors was misspelled. The correct name of the 8th author is Shuiwang Ji.

The Royal Society of Chemistry apologises for these errors and any consequent inconvenience to authors and readers.

